# Regulation of CCL5 Expression in Smooth Muscle Cells Following Arterial Injury

**DOI:** 10.1371/journal.pone.0030873

**Published:** 2012-01-23

**Authors:** Huan Liu, Huan Ning, Hongchao Men, Rong Hou, Mingui Fu, Hailin Zhang, Jianguo Liu

**Affiliations:** 1 Department of Pharmacology, Hebei Medical University, Shijiazhuang, China; 2 Heibei North University Medical College, Zhangjiakou, China; 3 Division of Infectious Diseases, Allergy and Immunology, Department of Internal Medicine, Saint Louis University School of Medicine, St. Louis, Missouri, United States of America; 4 Shock/Trauma Research Center & Department of Basic Medical Science, School of Medicine, University of Missouri Kansas City, Missouri, United States of America; University of Leuven, Rega Institute, Belgium

## Abstract

Chemokines play a crucial role in inflammation and in the pathophysiology of atherosclerosis by recruiting inflammatory immune cells to the endothelium. Chemokine CCL5 has been shown to be involved in atherosclerosis progression. However, little is known about how CCL5 is regulated in vascular smooth muscle cells. In this study we report that CCL5 mRNA expression was induced and peaked in aorta at day 7 and then declined after balloon artery injury, whereas IP-10 and MCP-1 mRNA expression were induced and peaked at day 3 and then rapidly declined.

The expression of CCL5 receptors (CCR1, 3 & 5) were also rapidly induced and then declined except CCR5 which expression was still relatively high at day 14 after balloon injury. In rat smooth muscle cells (SMCs), similar as in aorta CCL5 mRNA expression was induced and kept increasing after LPS plus IFN-gamma stimulation, whereas IP-10 mRNA expression was rapidly induced and then declined. Our data further indicate that induction of CCL5 expression in SMCs was mediated by IRF-1 via binding to the IRF-1 response element in CCL5 promoter. Moreover, p38 MAPK was involved in suppression of CCL5 and IP-10 expression in SMCs through common upstream molecule MKK3. The downstream molecule MK2 was required for p38-mediated CCL5 but not IP-10 inhibition. Our findings indicate that CCL5 induction in aorta and SMCs is mediated by IRF-1 while activation of p38 MAPK signaling inhibits CCL5 and IP-10 expression. Methods targeting MK2 expression could be used to selectively regulate CCL5 but not IP-10 expression in SMCs.

## Introduction

Atherosclerosis is generally recognized a chronic inflammatory disease characterized by arterial lesions composed of cholesterol, immune cells and fibrosis [1], [2]. Recruitment of inflammatory immune cells as well as migration and proliferation of vascular smooth muscle cells (VSMCs) in the wall of injured blood vessels play critical roles in the pathogenesis of atherosclerosis [3], [4]. Migration of immune cells and VSMCs to the intima is guided by chemokines secreted from immune cells and resident vascular cells which participate in the inflammatory process of atherosclerosis [5].

Chemokines are small soluble molecules that are best known for their potent abilities to induce cellular migration, particularly by leukocytes, during inflammation and infection [6], [7], [8]. Migration of leukocytes to the sites of infection is a critical step for initiating a proper immune response against invading pathogens. CCL5 (Regulated upon Activation, Normal T cell Expressed and Secreted, RANTES) is a member of the CC chemokine family which also includes monocyte chemoattractant protein (MCP)-1, MCP-2, MCP-3, and macrophage inhibitory protein (MIP)-1α, MIP-1β and I-309. CCL5 was originally discovered by subtractive hybridization in T but not in B cells and is expressed in platelets, macrophages, tubular epithelium, synovial fibroblasts, as well as selected tumor cells [9]. CCL5 plays an essential role in inflammation by recruiting T cells, macrophages, dendritic cells, eosinophils, NK cells, mast cells, and basophils to the sites of inflammation and infection [10], [11]. It has been reported that many chemokines, including CCL5 (RANTES) and CCL2 (MCP-1), are increased in vascular wall cells and cooperate in leukocyte recruitment to the injured artery during vascular remodeling [12]. Blockade of CCL5 binding to its receptor CCR5 by met-RANTES [13] and treatment with CCL5 variant [^44^AANA^47^]-RANTES [14] reduce atherosclerotic plaque formation or prevent progression of established atherosclerotic lesions in mice. Other studies further demonstrate that double deletion of CCR5 and ApoE in mice reduces the development of diet-induced atherosclerosis [15]. Taken together, all data indicate an important role for CCL5/CCR5 signaling pathway in the pathogenesis of atherosclerosis.

Regulation of CCL5 in immune cells has been well studied. It has been reported that transcription factors such as interferon regulatory factor-3, STAT1 and NF-κB mediate CCL5 transcription and production [16], [17]. Using an *Helicobacter pylori* infection model, Kudo et al showed that maximal *H. pylori*-induced CCL5 gene transcription required the presence of the interferon-stimulated response element (ISRE) and the cyclic AMP-responsive element [18]. Our previous studies further mapped the IFN-γ-inducible ISRE in the CCL5 promoter as IRF1-RE [19], [20]. In SMCs Y-box binding protein-1 (YB-1) has been reported to be able to regulate CCL5 expression in neointimal SMCs but not in macrophages, and deletion of YB-1 in carotid arteries inhibits CCL5 expression in SMCs and impairs neointima formation via reduced macrophage infiltration [21]. However, it is not fully understood how CCL5 is regulated in SMCs in response to inflammatory signaling.

We found in this study that chemokine CCL5, IP-10 and MCP-1 have different kinetics after balloon injury. In SMCs IFN-γ enhances LPS-induced CCL5 transcription and p38 MAPK signaling pathway inhibits CCL5 and IP-10 transcription via different downstream molecules.

## Results

### Different kinetics of chemokine expression in aorta after balloon artery injury

**Figure 1:**
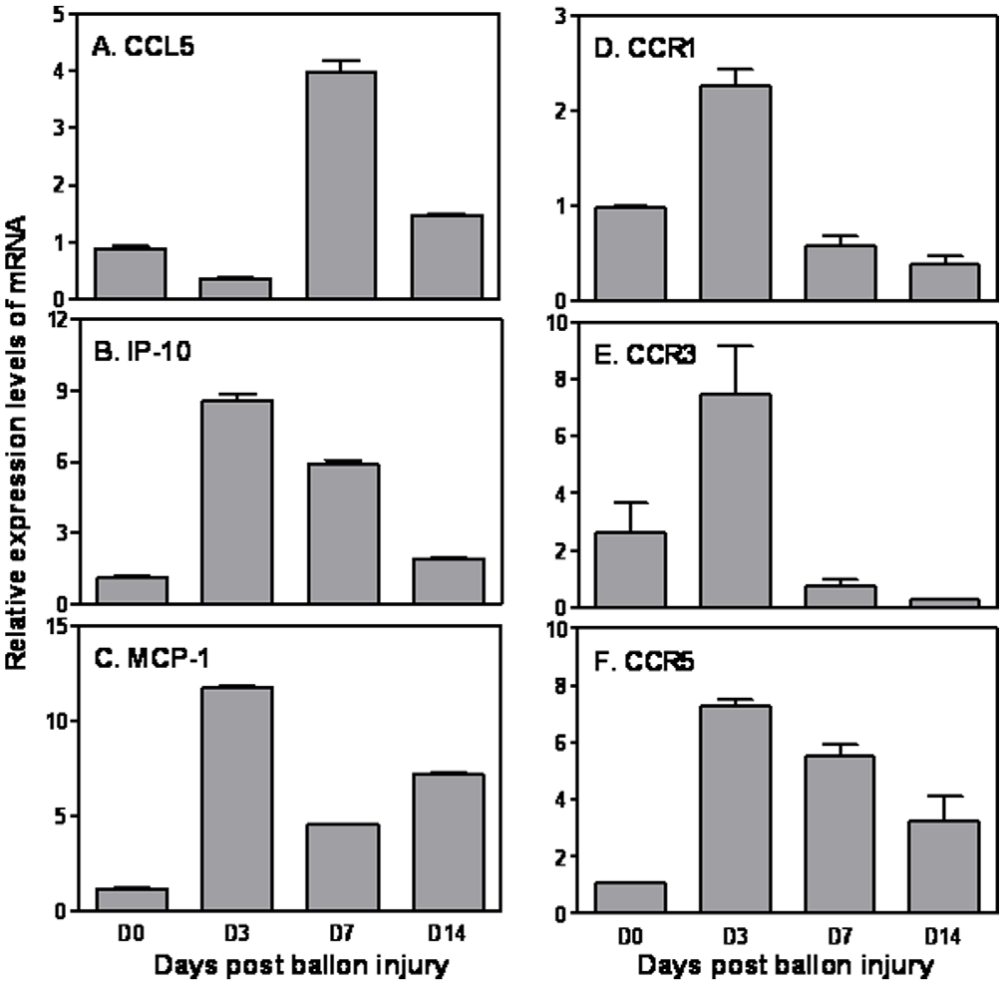
Kinetic expression of chemokines in aorta after balloon injury. Total RNA was extracted from aorta and used for real time quantitative PCR (qRT-PCR) measurement of CCL5 (A), IP-1 (B) and MCP-1 (C) mRNA expression as well as CCR1 (D), CCR3 (E) and CCR5 (F) mRNA expression. qRT-PCR data were normalized relative to GAPDH mRNA expression levels in each respective sample and further normalized to the results from the un-treated aorta, which was set as 1. Results shown are mean plus SD of three to four independent experiments.

To determine the expression profile of chemokines in aorta after injury, we measured the kinetic expression of CCL5, IP-10 and MCP-1 mRNA in aorta after balloon injury. CCL5 mRNA expression was slightly down-regulated at day 3 and then increased and peaked at day 7, followed by decline at day 14 after balloon injury (Fig. 1A). In contrast, IP-10 mRNA expression was rapidly induced and peaked at 3 days and than declined to a level similar to uninjured aorta at day 14 after balloon injury (Fig. 1B). MCP-1 mRNA expression was also rapidly induced by balloon injury followed by decline but kept at relatively high level at 14 days after injury (Fig. 1C). The different kinetics of chemokine CCL5, IP-10 and MCP-1 expression after artery injury suggest that different chemokines may play different roles in attracting different types of host cells to the local environment to participate in generation of local inflammation. To determine the expression levels of CCL5 receptors, we measured the expression of CCR1, CCR3 & CCR5 in aorta after balloon injury. The expression of all three CCL5 receptors (CCR1, 3 & 5) was rapidly induced at day 3 and then declined except CCR5 (the major receptor for CCL5) which, though gradually declined, but still remained at a relatively high level compared to other CCL5 receptors (Fig. 1D–F), suggesting that CCR5 may play an important role in CCL5-mediated chronic inflammatory responses in aorta.

### Vascular smooth muscle cells express chemokine CCL5, IP-10 and MCP-1 in response to inflammatory stimuli

**Figure 2:**
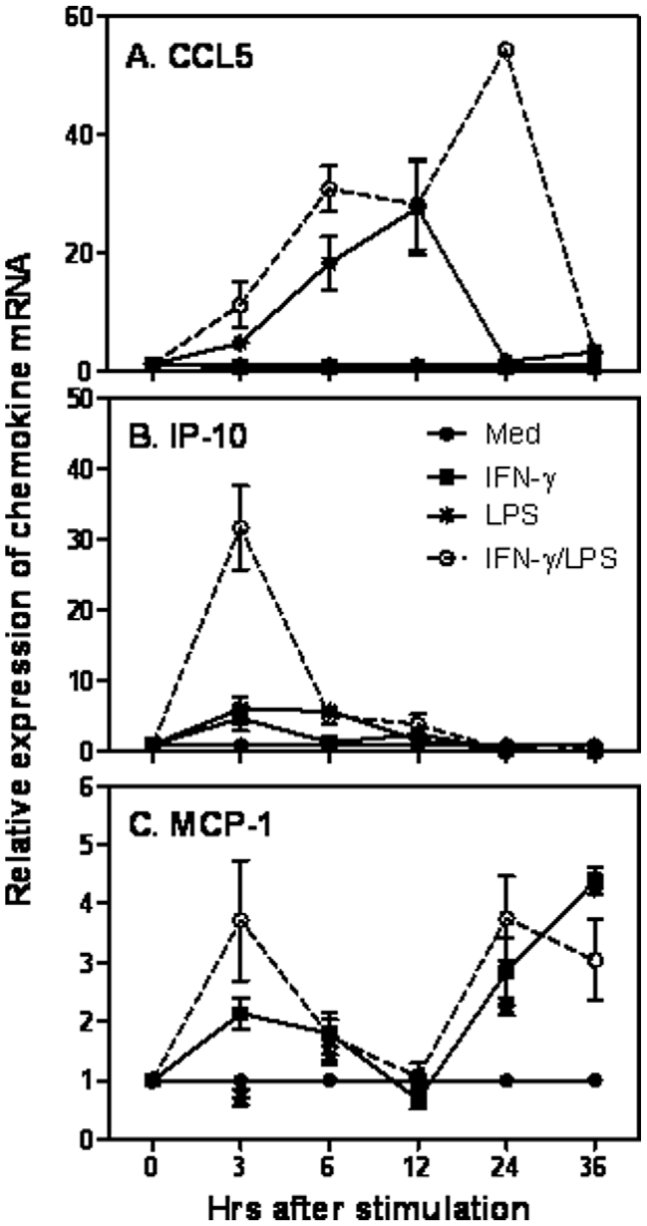
Kinetic expression of chemokines in rat vascular smooth muscle cells. Total RNA was extracted from rat smooth vascular muscle cells treated with IFN-γ, LPS, or IFN-γ plus LPS for different times as indicated and reverse transcribed into cDNA for measurement of CCL5 (A), IP-10 (B) and MCP-1 (C) expression by qRT-PCR. Data were normalized relative to GAPDH mRNA expression levels in each respective sample and further normalized to the results from the un-treated group, which was set as 1.

The increased expression of chemokine CCL5, IP-10 and MCP-1 in aorta after balloon injury compelled us to investigate whether vascular smooth muscle cells (VSMCs) could express these chemokines. We isolated and cultured primary rat VSMCs, and stimulated the cells with LPS and IFN-γ for different times, with a purpose of LPS stimulation representing activation of innate signaling TLR4, and IFN-γ produced mainly from NK cells, CD8^+^ T cells and CD4^+^ T helper 1 (Th1) cells representing signals derived from host immune responses against inflammation. Rat CCL5 mRNA expression was hardly induced by IFN-γ alone whereas strongly induced by LPS, and reached a peak at 12 hours after LPS stimulation, and co-stimulation of rat VSMCs with LPS and IFN-γ synergistically enhanced CCL5 mRNA expression with a peak at 24 hours (Fig. 2A), suggesting a secondary response might be involved in CCL5 expression at the late phase. Consistent with the *in vivo* data, IP-10 mRNA expression was rapidly induced and peaked at 2–3 hrs after LPS and IFN-γ stimulation and then rapidly declined (Fig. 2B). However, in difference from CCL5 and MCP-1, either LPS or IFN-γ alone could not induce a strong IP-10 mRNA expression (Fig. 2B). To our surprise the expression of MCP-1 had two peaks with the early one at 3 hours and the late one around 24 hours (Fig. 2C), indicating the presence of primary and secondary responses of MCP-1 expression after arterial injury. Further investigation is desired to explore the molecular mechanisms of these different responses of MCP-1 expression after arterial injury. Nevertheless, these data suggest that the expression patterns of CCL5, IP-10 and MCP-1 mRNA are similar between aorta and VSMCs.

### IRF-1 mediates CCL5 mRNA expression in VSMCs

**Figure 3:**
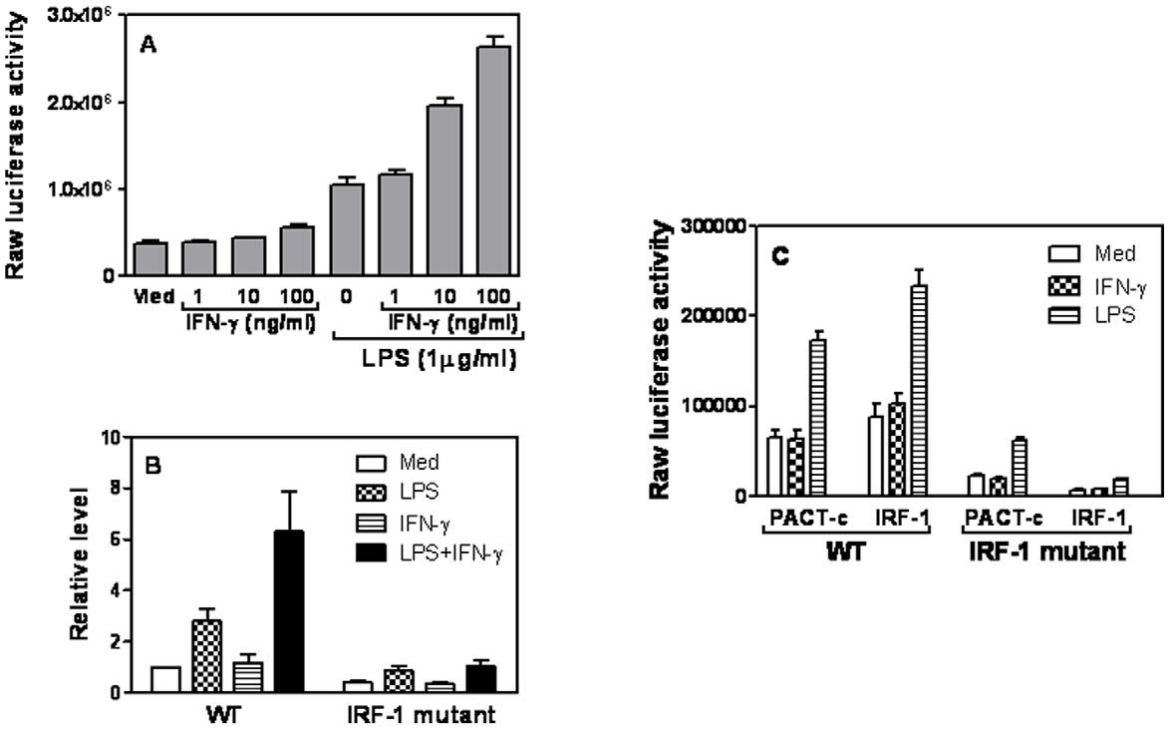
IRF-1 mediates CCL5 transcription in smooth muscle cells. (A) 5 µg of CCL5 promoter driven luciferase construct was transfected into A7r5 cells by Lipofectamin2000. The transfected cells were treated with different amounts of IFN-γ as indicated, LPS (1 µg/ml), or IFN-γ plus LPS for 7 hours, followed by cell lysis and detection of luciferase activity. (B) 5 µg of WT and IRF-1 mutant CCL5 promoter constructs were respectively transfected into A7r5 cells by Lipofectamine2000 and treated with IFN-γ (10 ng/ml) and LPS (1 µg/ml) for 7 hrs, followed by measurement of luciferase activity in cell lysates. Luciferase activities were normalized to the activities obtained in WT CCL5 promoter transfected cells without treatment which was set as 1. (C) 5 µg of WT and IRF-1 mutant CCL5 promoter constructs were respectively co-transfected with PACT-c (empty vector) and IRF-1 expression plasmid into A7r5 cells by Lipofectamine2000, followed by IFN-γ and LPS treatment. Luciferase activity was measured in cell lysates. All results shown represent mean plus SD of three to four separate experiments.

Migration of inflammatory cells to the injured sites needs constant guidance of chemokines. Since CCL5 mRNA expression was comparable between aorta and VSMCs and remained at a relatively high level 7 days after injury, we focused our study on how CCL5 expression is regulated. Next we wanted to know whether IFN-γ and LPS could induce CCL5 promoter activation in VSMCs since steady state CCL5 mRNA expression is mainly determined by transcription. As shown in figure 3A, IFN-γ alone had little effect on CCL5 promoter activation whereas LPS induced more than twofold of CCL5 promoter activation compared with untreated cells. Moreover, addition of IFN-γ dose-dependently enhanced LPS-induced CCL5 promoter activation. Since our previous studies demonstrate that IRF-1 plays an important role in CCL5 expression in macrophages [19], we wondered that IRF-1 may also control CCL5 expression in VSMCs. We transiently transfected the wild type and IRF-1-mutant CCL5 promoter into A7r5 cells and treated the cells with IFN-γ and LPS, followed by measurement of luciferase activity in cell lysates. Consistent with the above data, IFN-γ and LPS synergistically enhanced the wild type CCL5 promoter activity whereas the synergistic effect was almost completely lost when IRF-1 mutant CCL5 promoter used (Fig. 3B), indicating the importance of IRF-1 in regulation of CCL5 expression in SMCc. Compared with empty-vector transfected cells overexpression of IRF-1 enhanced CCL5 promoter activity in A7r5 cells transfected with WT but not IRF-1 mutant CCL5 promoter (Fig. 3C). Taken together, these data indicate that transcription factor IRF-1 mediates CCL5 expression via the IRF-1 response element in CCL5 promoter in VSMCs in response to LPS and IFN-γ stimulation.

### IRF-1 binds to the IRF response element in CCL5 promoter

**Figure 4:**
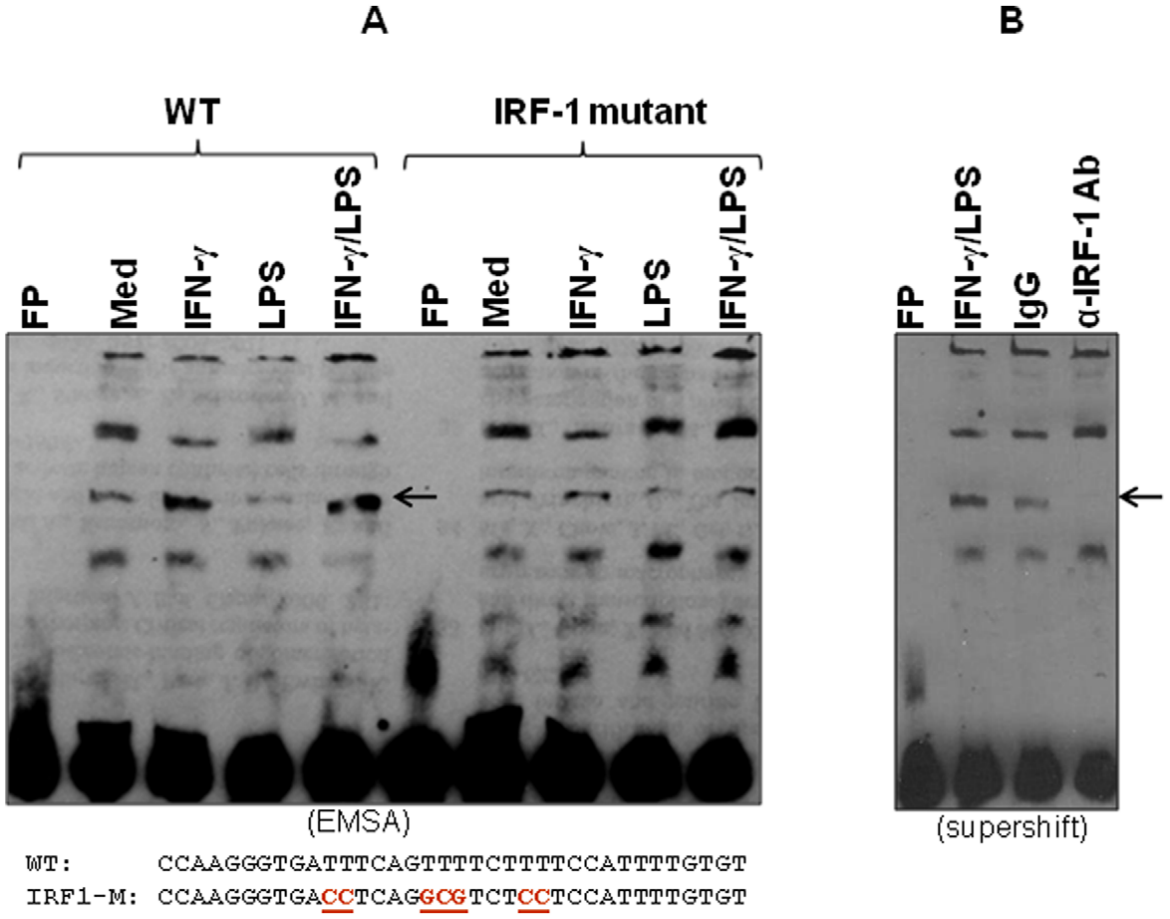
IRF-1 specifically binds to the CCL5 promoter. (A) Nuclear extracts were isolated from A7r5 cells following IFN-γ or LPS stimulation for 4 h. EMSA was performed with 10 µg of nuclear extract for each sample and a double-stranded oligonucleotide probe containing the −161/−134 region of the CCL5 promoter (sequence given with the critical IRF-RE underlined). (B) “Supershift” EMSA was performed with the −161/−134 probe and nuclear extracts from IFNγ/LPS-stimulated macrophages with an anti IRF-1 antibody and its control rabbit IgG being used. The IRF-1-related complex is indicated by an arrow.

To determine whether IRF-1 bound to CCL5 promoter, we performed EMSA using nuclear extracts isolated from A7r5 cells stimulated with IFN-γ or LPS. As shown in figure 4A, there were several nuclear DNA-binding complex formed with an oligonucleotide containing the IRF-1 response element in CCL5 promoter, and the intensity of one binding complex was increased upon IFN-γ or IFN-γ plus LPS stimulation (indicated by an arrow). This increased binding intensity was reduced to basal level with IRF-1 mutated probe, indicating the sequence specify of the binding to CCL5 promoter. To further confirm IRF-1 indeed binds to CCL5 promoter, we performed “supershift” EMSA using an anti-IRF-1 antibody. The increased binding upon IFN-γ plus LPS treatment was completely supershifted by adding anti-IRF-1 antibody but not control IgG (Fig. 4B), confirming that the increased binding complex upon IFN-γ and LPS stimulation indeed contains IRF-1.

### p38 MAPK inhibits CCL5 expression in VSMCs

**Figure 5:**
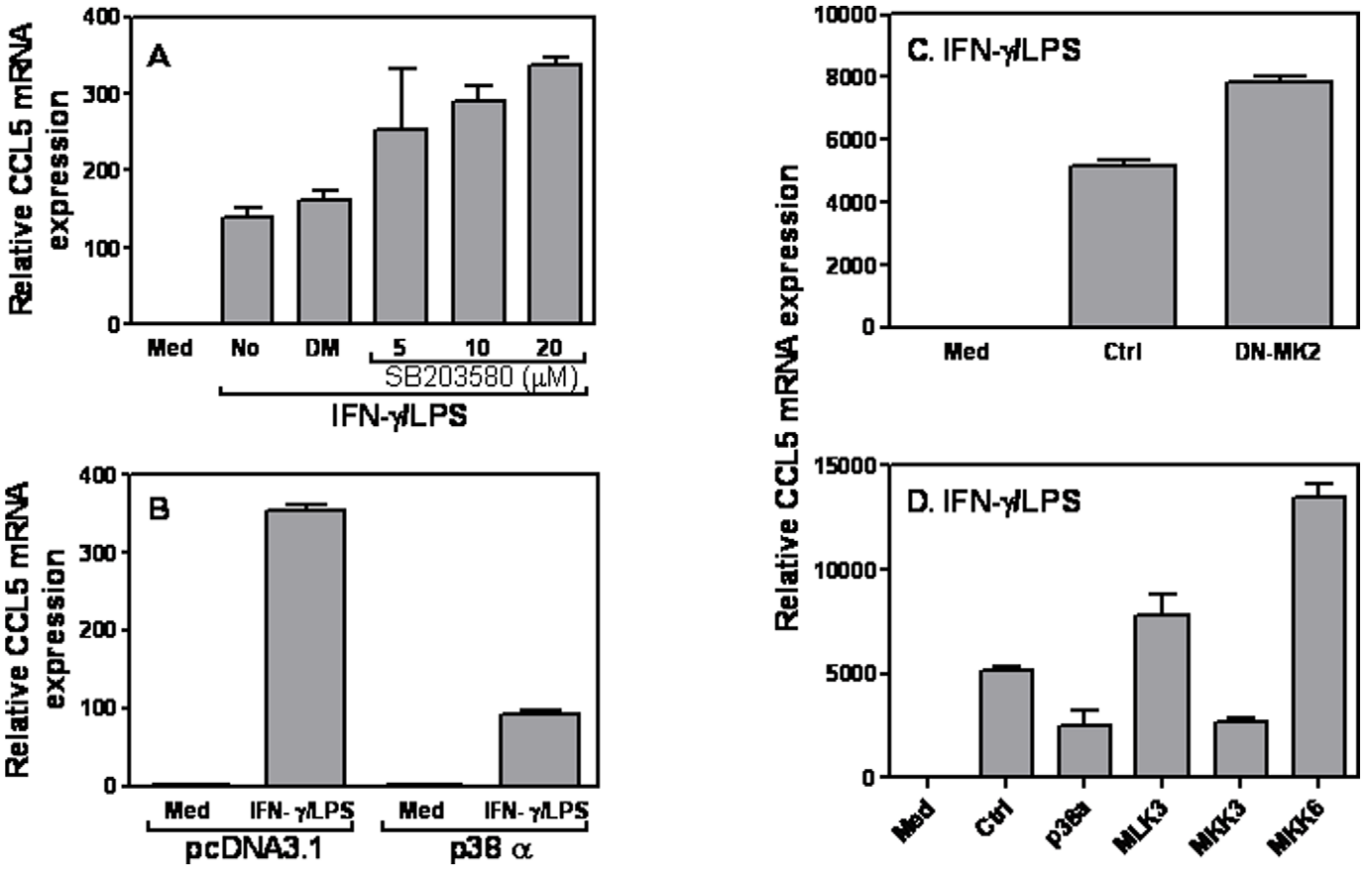
The role of p38 MAPK in CCL5 expression. (A) 1×10^6^ A7r5 cells were treated with different amounts of a p38 MAPK antagonist SB203580 for 1 h prior to addition of various stimuli as indicated. An identical amount of dissolvent DMSO was used for negative controls. 6 hrs later, total RNA was extracted for measurement of CCL5 mRNA expression by qRT-PCR. 5 µg of different expression plasmids encoding different molecules involved in the p38 MAPK signaling pathway including p38α (B), and DN-MK2 (C), as well as MLK3, MKK3, and MKK6 (D) or empty vector were transiently transfected with Lipofectamine2000. 48 h after transfection, the cells were treated with LPS (1 µg/ml) plus IFN-γ (10 ng/ml) for 6 h, followed by measurement of CCL5 mRNA expression by qRT-PCR. qRT-PCR data were normalized relative to GAPDH mRNA expression levels in each respective sample and further normalized to the results from the un-treated group, which was set as 1. Results shown are mean plus SD of three independent experiments.

It has been reported that many cytokines, including IL-12, IL-1β, IL-6, TNF-α and IL-10, play important roles in immune responses and are regulated by p38 MAPK [22], [23]. To investigate whether CCL5 expression was regulated by p38 MAPK, we first blocked p38 MAPK with SB203580 prior to IFN-γ and LPS stimulation, followed by measurement of CCL5 mRNA expression. Blockade of p38 pathway with SB203580 dose-dependently increased CCL5 mRNA expression in A7r5 SMCs compared with DMSO-treated cells (Fig. 5A). In line with this finding, overexpression of p38 MAPK significantly inhibited CCL5 mRNA expression in cells treated with IFN-γ and LPS (Fig. 5B), indicating that p38 MAPK signaling pathway suppresses CCL5 expression in VSMCs in response to IFN-γ and LPS stimulation. To dissect which component in p38 pathway cascade was involved in inhibition of CCL5 mRNA expression, we first transfected a dominant negative mutant of MK2 (DN-MK2) or empty vector into A7r5 cells, followed by IFN-γ and LPS treatment. Blocking MK2 by DN-MK2 increased CCL5 mRNA expression (Fig. 5C), suggesting that p38 acts through downstream molecule MK2 to inhibit CCL5 expression. To further determine which upstream molecule in p38 pathway cascade was involved in CCL5 inhibition, we individually transfected MLK3, MKK3 and MKK6 or empty vector into A7r5 cells followed by IFN-γ and LPS stimulation. As shown in Fig. 5D, two upstream molecules MLK3 and MKK6 enhanced IFN-γ- and LPS-induced CCL5 mRNA expression whereas MKK3 inhibited it, indicating that the upstream molecule MKK3 and downstream molecule MK2 are participated in p38-medited CCL5 mRNA inhibition.

### p38 MAPK inhibits IP-10 expression in SMCs

**Figure 6:**
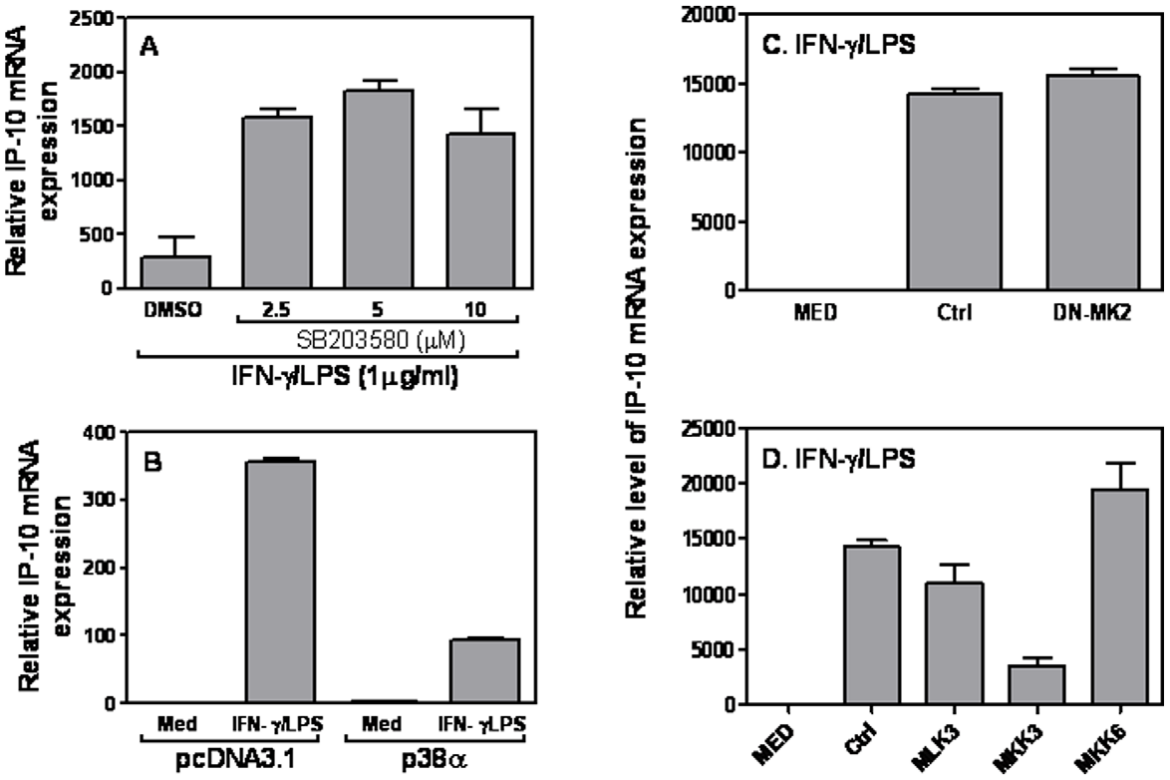
The role of p38 MAPK in IP-10 expression. (A) 1×10^6^ A7r5 cells were treated with different amounts of a p38 MAPK antagonist SB203580 for 1 h prior to addition of various stimuli as indicated. An identical amount of dissolvent DMSO was used for negative controls. 6 hrs later, total RNA was extracted for measurement of IP-10 mRNA expression by qRT-PCR. 5 µg of different expression plasmids encoding different molecules involved in the p38 MAPK signaling pathway including p38α (B), and DN-MK2 (C), as well as MLK3, MKK3, and MKK6 (D) or empty vector were transiently transfected with Lipofectamine. 48 h after transfection, the cells were treated with LPS (1 µg/ml) plus IFN-γ (10 ng/ml) for 6 h, followed by measurement of IP-10 mRNA expression by qRT-PCR. qRT-PCR data were normalized relative to GAPDH mRNA expression levels in each respective sample and further normalized to the results from the un-treated group, which was set as 1. Results shown are mean plus SD of three independent experiments.

To determine whether p38 MAPK was also involved in control of IP-10 mRNA expression, we blocked p38 with SB203580 prior to IFN-γ and LPS stimulation, followed by measurement of IP-10 mRNA expression as described above. Blockade of p38 pathway with SB203580 increased IP-10 mRNA expression in VSMCs compared with DMSO-treated cells (Fig. 6A). Consistently, overexpression of p38 MAPK inhibited IP-10 mRNA expression (Fig. 6B), indicating a similar regulation of IP-10 expression as the CCL5 by p38 signaling in VSMCs. In difference from CCL5, blocking MK2 by dominant negative mutant DN-MK2 did not affect CCL5 expression (Fig. 6C) and overexpression of MKK3 but not MLK3 and MKK6 inhibited IP-10 mRNA expression (Fig. 6D), suggesting that MKK3 but not MK2 is involved in p38-mediated IP-10 inhibition in VSMCs.

## Discussion

Chronic inflammation plays an important role in the pathogenesis of atherosclerosis. One of the critical steps for initiation of inflammation is recruitment of immune cells such as T cells, NK cells and monocytes to atherosclerotic lesions under the guidance of chemokines. High levels of chemokines secreted around vascular cells within the lesions determine which types of cells could migrate to the lesion. Our data indicate that multiple chemokines were induced in aorta after balloon artery injury (Fig. 1). Interestingly, chemokine CCL5 and IP-10 showed a different kinetics in aorta following injury in that IP-10 was fast induced and reached a peak at day 3 whereas CCL5 was induced at a late stage and reached the peak at day 7. This differential kinetics suggests that during inflammation each chemokine may participate in directing specific type of cells to migrate to atherosclerotic lesions. It has been reported that MCP-1 mediates transendothelial migration but not shear-resistant arrest of monocytes on activated endothelial cells [24] and IP-10 has been described as a chemoattractant for monocytes and activated lymphocytes [25]. The association of CCL5 induction with leukocyte recruitment in the injured arteries has been recently demonstrated by Boehm's group. Using CCL5 knockout mice they have demonstrated that local induction of CCL5 in medial VSMCs regulates CD3^+^ T cell and macrophage infiltration and late neointimal formation [26]. We did not measure serum CCL5 levels in this study because the connection between serum CCL5 levels and the presence of coronary heart disease is controversial [27], [28]. Moreover, local CCL5 expression has been shown to closely associate with chronic inflammation in atherosclerosis [26]. Platelet-derived CCL5 deposits on endothelial cells to induce monocyte arrest and promote atheroscrosis [29], [30], further indicates the role for local CCL5 in facilitation of local inflammation. It will be interested to investigate the correlation of serum CCL5 levels to the degrees of local arterial inflammation in future study.

Interferons, including type I and type II IFNs, are cytokines that effectively enhance host defenses against various infections and immunosurveillance against tumors [31]. IFN-γ, the only member of the type II IFN family, is produced by a variety of cell types, including NK cells, NK T cells, CD4^+^ and CD8^+^ T cells as well as SMCs. IFN-γ is essential for mounting both innate and cellular immune responses. It also plays an important role in the chronic nature of inflammatory responses associated with atopic dermatitis, rheumatoid arthritis, and systemic lupus erythematosus [31]. IFN-γ acts on a remarkable range of distinct cell populations including macrophages, natural killer cells, B cells, ECs and VSMCs. It has been shown that IFN-γ is necessary and sufficient to cause vascular remodeling in a mouse model of atheroma, as the serological neutralization or genetic absence of IFN-γ markedly reduces the extent of atherosclerosis [32]. IFN-γ is thought to participate in promoting atherogenic responses through transcription factor STAT1, which plays a major role in driving the pro-inflammatory responses leading to EC dysfunction, VSMC proliferation and vascular damage [33]. Our data suggest that IFN-γ can promote local inflammatory responses through induction of CCL5 via IRF-1 in VSMCs.

TLR4 is the first toll-like receptor (TLR) being discovered in the TLR family. TLR4 is expressed on a variety of cells, including ECs and VSMCs, and thus initiates and sustains the inflammatory response in atherosclerosis [34]. TLR4 signaling leads to the induction of various target genes, including type I IFNs, pro-inflammatory cytokines, chemokines and cell surface molecules [35], [36]. At the sites of tissue damage or excessive tissue remodeling, such as atherosclerotic lesions, endogenous ligands for TLR4, including HSP, OxLDL and HMGB1, etc, are accumulated in the local environment that play a direct role in promoting inflammation [37]. Using LPS and IFN-γ to mimic the endogenous innate and adaptive immune signals, we found that in difference from IP-10 which requires both IFN-γ and LPS signaling for a strong induction in rat SMCs (Fig. 2B), CCL5 could be induced by LPS alone and further increased by addition of IFN-γ at a later time point (Fig. 2A), whereas MCP-1 could be induced by either IFN-γ or LPS signaling (Fig. 2C). The expression pattern of IP-10 is different from a previous report that IP-10 is gradually induced till day 14 in rat carotid artery following balloon angioplasty [38]. We reason that this difference might be due to different procedures of angioplasty or different methods for detection of mRNA expression.

IFN-γ dose-dependently increased CCL5 promoter activation in VSMCs treated with LPS (Fig. 3). Our data further demonstrated that like in macrophages transcription factor IRF-1 mediates CCL5 transcription via the IRF-1 binding site in CCL5 promoter (Figs. 3 & 4). The IRF family consists of nine transcription factors that commonly possess a unique helix-turn-helix DNA-binding motif at the N-terminus and an activation domain in the C-terminus [39]. IRF-1, the first member of the IRF family to be identified, targets different sets of genes in various cell types in response to diverse cellular stimuli and evokes appropriate innate and adaptive immune responses [39]. Our previous studies demonstrated that IFN-γ-induced IRF-1 differentially regulates IL-12 p35 and p40 gene expression in macrophages [40]. IRF-1 has been well established as a critical effector molecule in IFN-γ-mediated signaling and in the development and function of NK cells, NK T cells and cytotoxic T lymphocytes [41], [42], [43], [44], [45], [46]. In this study our data indicate that IRF-1 also plays an important role in induction of CCL5 expression in VSMCs.

MAPK signaling pathway is involved in regulation of many cytokine and chemokine expression during inflammation. Since VSMCs are different from macrophages in CCL5 expression, in that the maximal CCL5 expression in VSMCs requires both IFN and LPS signaling whereas in macrophages LPS alone is sufficient, we decided to investigate the effects of MAPK on CCL5 expression in response to LPS and IFN-γ stimulation. Both blocking and overexpression data indicate that p38 MAPK triggered by LPS and IFN-γ inhibited CCL5 (Fig. 5) and IP-10 (Fig. 6) expression in VSMCs. When we looked more details to which molecule in the cascade actually was involved in signaling transduction, it turned out that MK2 mediated inhibition of CCL5 (Fig. 5C) but not IL-10 (Fig. 6C) in VSMCs in response to LPS and IFN-γ stimulation, whereas the upstream molecule MKK3 mediated both inhibition (Figs. 5D & 6D). These data suggest that methods targeting MK2 expression could be used to selectively regulate CCL5 but not IP-10 expression in SMCs, which may have potential therapeutic effects on preventing chronic inflammation in the blood vessel.

## Materials and Methods

### Rat carotid artery balloon injury

All procedures were in accordance with institutional guidelines approved by the Animal Care and Ethical Committee of Hebei Medical University (Shijiazhuang, China) under policies adhering to IASP guidelines for use of animals (the approval ID is 000172). Male Sprague-Dawley rats weighing 350 to 400 g were anesthetized with ketamine (40 mg/kg) and xylazine (5 mg/kg). The left common carotid artery was denuded of endothelium and stretched by three passages of a 2F embolectomy catheter according to standard protocols [47], [48]. At 3, 7, and 14 days after injury, animals were euthanized; both right (uninjured control) and left (injured) carotid arteries were harvested and snap-frozen in liquid nitrogen for RNA extraction.

### Cells

Arterial smooth muscle cells were harvested from the aortas of male Sprague-Dawley rats (200 to 250 g, Charles River Laboratories) by enzymatic dissociation [48], [49] The cells were cultured in DMEM supplemented with 10% FCS (Sigma, St. Louis, MO, endotoxin NMT 10.0 EU/ml), 100 U/mL penicillin, 100 µg/mL streptomycin, and 10 mmol/L HEPES (pH 7.4) (Sigma Chemical Co). Rat vascular smooth muscle cells (VSMCs) were passaged every 3 to 5 days, and experiments were performed on cells four to six passages from primary culture. The rat aortic smooth muscle cell line (A7r5 cells) was obtained from American Type Culture Collection, and maintained in DMEM supplemented with 2 mM glutamine, 100 units/ml of penicillin and streptomycin and 10% FBS (Sigma, St. Louis, MO, endotoxin NMT 10.0 EU/ml).

### Plasmids

Mouse CCL5 promoter-driven luciferase plasmids were generated previously [19]. Expression vectors pAct-1 (IRF-1), and control pAct-C were originally provided by Dr. T. Taniguchi (University of Tokyo, Japan). MAPK expression plasmids encoding MLK3, MKK3, MKK6, p38, dominant negative mutant MK2 and p38α were provided by Dr. Aihao Ding (Weill Cornell Medical College). All plasmid DNA were prepared with QIAGEN Endo-free Maxi-Prep kits.

### Reagents

LPS from *Escherichia coli* 0217:B8 were purchased from Sigma-Aldrich (St. Louis, MO). Recombinant mouse IFN-γ was purchased from Genzyme (Boston, MA). p38 MAPK antagonist SB203580 was purchased from Calbiochem (San Diego, CA).

### Quantitative real-time PCR

Reverse-transcription reactions were carried out as previously described [50], Quantitative real time PCR (qRT-PCR) was performed by a modified protocol. Briefly, cDNA samples converted from 1 µg of total RNA were diluted and studied at several concentrations. Diluted cDNA was mixed with a pair of primers (10 µM) targeting rat CCL5, IP-10, MCP-1 or GAPDH cDNA sequences, with SYBR green PCR master mix (Sigma-Aldrich, St. Louis) in a 15 µl volume. The following primers were used for PCR amplification of the rat CCL5 cDNA: sense: gcagctgcatccctcaccgt, antisense: gcagcagggagtggtgtccg; rat IP-10 cDNA: sense: cgccacagtgctgagaccga, antisense: caccctgtagtgggagtagcagct; rat MCP-1 cDNA sense; tgtcacgcttctgggcctgt, antisense: agcagcaggtgagtggggca; and rat GAPDH cDNA: sense: gacatg ccgcctggagaaac; antisense: agcccaggatgccctttagt.

### Transfection assay

Transient transfections were performed according to manufacture's protocol (Invitrogen). Briefly, for each condition 3×10^6^ A7r5 cells were transiently transfected with 4 µg of DNA plasmids (including reporter, effector, internal control and carrier DNA) by Lipofectamin2000. The transfected cells were cultured in RPMI1640 complete medium containing 10% FBS and incubated for 48 h prior to performing experiments. To measure luciferase activity, cells were pelleted and resuspended in lysis buffer. Luciferase activity was measured in cell lysates.

### Nuclear extract preparation

Nuclear extracts for EMSA were prepared according to the methods of Schreiber et al. [51]. Briefly, 5×10^7^ A7r5 cells were washed and resuspended in 400 µl buffer containing 10 mM HEPES, pH 7.9, 10 mM KCl, 0.1 mM EDTA, 1 mM DTT, 0.5 mM PMSF for 15 min on ice. Cells were lysed in 0.6% NP-40 with inversion for 10 sec. The homogenate was centrifuged for 5 min and the nuclear pellet was resuspended in ice-cold buffer containing 20 mM HEPES, pH 7.9, 0.4 M NaCl, 1 mM EDTA, 1 mM DTT, 1 mM PMSF at 4°C for 30 min with rotating. Following centrifugation for 10 min, the supernatant was either used immediately or frozen at −70°C.

### LightShift Chemiluminescent EMSA

LightShift Chemiluminescent EMSA was performed according to the manufacture's protocol (Pierce, Rockford, IL). Briefly, single strand wild type and IRF-1 mutant oligonucleotides were labeled with biotin using a Biotin 3′ End DNA Labeling Kit (Pierce, Rockford, IL). Equal amounts of labeled and complementary oligos were annealed to prepare double-strand wild type and mutant probes by heating at 90°C for 2 min, followed by slowly cooling to room temperature for 30 min. The labeled double-strand probes were mixed with 10 µg crude nuclear extracts and incubated at room temperature for 20–30 min in the presence of 1 µg poly(dI-dC). The mixture was then fractionated through a 5% native polyacrylamide gel in 0.5×TBE buffer for about 1 h at 100 V. The gel was transferred to positively charged nylon membrane at 4°C for 1 h at 100 V. When transfer was completed, the membrane was cross-linked at 120 mJ/cm^2^ using a commercial UV-light cross-linker instrument equipped with 254 nm bulbs. Then, the membrane was blocked and applied with Streptavidin-Horseradish Peroxidase Conjugate (1∶300 dilution) for 15 min, followed by thoroughly washing, and exposed to X-ray film after incubation with the chemiluminescence reagents. The X-ray film was developed according to the manufacture's instruction.

### Statistical analysis

Student *t* test was performed wherever applicable. Standard deviation of the mean is shown unless otherwise indicated. *, *p*<0.05; **, *p*<0.01; ***, *p*<0.001.
